# Selected Trace Elements and Their Impact on Redox Homeostasis in Eye Health

**DOI:** 10.3390/biom14111356

**Published:** 2024-10-24

**Authors:** Joanna Wróblewska, Jarosław Nuszkiewicz, Marcin Wróblewski, Weronika Wróblewska, Alina Woźniak

**Affiliations:** 1 Department of Medical Biology and Biochemistry, Faculty of Medicine, Ludwik Rydygier Collegium Medicum in Bydgoszcz, Nicolaus Copernicus University in Toruń, 24 Karłowicza St., 85-092 Bydgoszcz, Poland; joanna.wroblewska@cm.umk.pl (J.W.); marcin.wroblewski@cm.umk.pl (M.W.); 2 Student Research Club of Medical Biology and Biochemistry, Department of Medical Biology and Biochemistry, Faculty of Medicine, Ludwik Rydygier Collegium Medicum in Bydgoszcz, Nicolaus Copernicus University in Toruń, 85-092 Bydgoszcz, Poland; 316714@stud.umk.pl

**Keywords:** antioxidants, copper, eye, iron, oxidative stress, redox balance, selenium, trace elements, vision health, zinc

## Abstract

Oxidative stress plays a crucial role in the pathogenesis of various ocular degenerative diseases, leading to structural and functional changes in eye tissues. This imbalance between reactive oxygen species (ROS) and antioxidants significantly contributes to conditions such as age-related macular degeneration, diabetic retinopathy, cataracts, and glaucoma. Both enzymatic and nonenzymatic antioxidants are vital for maintaining ocular health by neutralizing ROS and restoring cellular redox balance. Essential trace elements, including iron, zinc, copper, and selenium, are fundamental for the proper functioning of these antioxidant systems. Iron is indispensable for enzymatic activity and cellular energy production, zinc supports numerous proteins involved in visual functions and antioxidant defense, copper is essential for various enzymatic reactions preventing oxidative stress, and selenium is critical for the activity of antioxidant enzymes such as glutathione peroxidase (GPX) and thioredoxin reductase (TrxR). This review summarizes current research on the complex interactions between oxidative stress and trace elements in ocular diseases, highlighting the therapeutic potential of antioxidant supplementation to mitigate oxidative damage and improve eye health. By integrating insights from studies on oxidative stress, trace elements, and eye physiology, this article underscores new diagnostic and therapeutic strategies that could lead to more effective prevention and treatment of ocular diseases, aiming to enhance clinical outcomes and guide future research in optimizing therapeutic strategies for eye health.

## 1. Introduction

Oxidative stress plays a critical role in the pathogenesis of various eye degenerative diseases, causing damage to ocular tissues. This damage encompasses alterations in tissue structure and function, heightened vascular permeability, microvascular irregularities, and the initiation of neovascularization processes. Consequently, these changes may lead to the formation of lesions in the cornea, conjunctiva, and optic nerve, as well as to the denaturation of lens crystallins, elevations in intraocular pressure, and degeneration of the retina [1]. This oxidative burden is particularly notable in significant retinal disorders such as age-related macular degeneration, diabetic retinopathy, degenerative myopia, inherited retinal dystrophies, and retinal detachment [2]. However, its impact is not limited to retinal afflictions but encompasses a broad spectrum of ocular conditions, including Dry Eye Disease (DED), cataracts, keratoconus, Fuchs’ endothelial dystrophy, bullous keratopathy, and glaucoma [3]. In cells, a finely tuned balance of antioxidants regulates both reactive oxygen species (ROS) and reactive nitrogen species (RNS), some of which are free radicals.

The protective mechanisms against oxidative stress in ocular tissues include both enzymatic and nonenzymatic antioxidants [1]. Enzymatic antioxidants such as superoxide dismutase (SOD), glutathione peroxidase (GPX), catalase (CAT), and bilirubin neutralize oxidative species directly, while essential enzymes such as glutathione reductase (GR), thioredoxin (Trx), and thioredoxin reductase (TrxR) restore critical thiols in proteins to maintain cellular redox balance. Nonenzymatic antioxidants, including vitamins E, A, and C, flavonoids, carotenoids, glutathione (GSH), plant polyphenols, uric acid, theaflavin, allyl sulfides, curcumin, melatonin, bilirubin, polyamines, and antioxidant elements (e.g., zinc, copper, and selenium), support this defense [4,5,6]. Iron, zinc, copper, and selenium are essential trace elements that play a key role in eye physiology and are crucial for the proper functioning of antioxidant enzymes [7,8]. As antioxidant levels decrease with age, oxidative stress rises, causing damage throughout the eye [9]. Approaches to reduce oxidative stress involve administering antioxidants to neutralize ROS and adjusting cellular signaling pathways that govern ROS generation and antioxidant defense mechanisms.

This review aims to summarize current research on the complex interactions between oxidative stress and trace elements in ocular diseases. It emphasizes the potential therapeutic benefits of antioxidant supplementation to mitigate oxidative damage and improve eye health. By integrating insights from studies on oxidative stress, trace elements, and eye physiology, this article seeks to highlight new diagnostic and therapeutic strategies that could lead to more effective prevention and treatment of ocular diseases.

This review hypothesizes that oxidative stress plays a central role in the development of ocular degenerative diseases and that key trace elements—iron, zinc, copper, and selenium—are critical in modulating this oxidative stress through their involvement in antioxidant defense mechanisms. We further hypothesize that disruptions in the balance of these trace elements contribute to the onset and progression of eye diseases by impairing redox homeostasis. Additionally, we propose that targeted antioxidant supplementation aimed at restoring this balance may mitigate oxidative damage and improve ocular health outcomes.

To conduct this review, we performed a comprehensive literature search across multiple databases, including PubMed, Google Scholar, and Web of Science. The search terms included “oxidative stress”, “ocular diseases”, “iron”, “zinc”, “copper”, and “selenium”. The search was restricted to articles published in English or Polish. Studies were included if they met the following criteria: the population consisted of human subjects or relevant animal models; the focus was on the role of trace elements in oxidative stress and ocular health; and the study design encompassed original research articles, reviews, and meta-analyses. Studies were excluded if they did not address the interaction between oxidative stress and trace elements or lacked peer review. Key findings related to the impact of trace elements on oxidative stress and ocular health were systematically identified and synthesized.

## 2. The Oxidant–Antioxidant Balance and Oxidative Stress in the Eye

**Figure 1:**
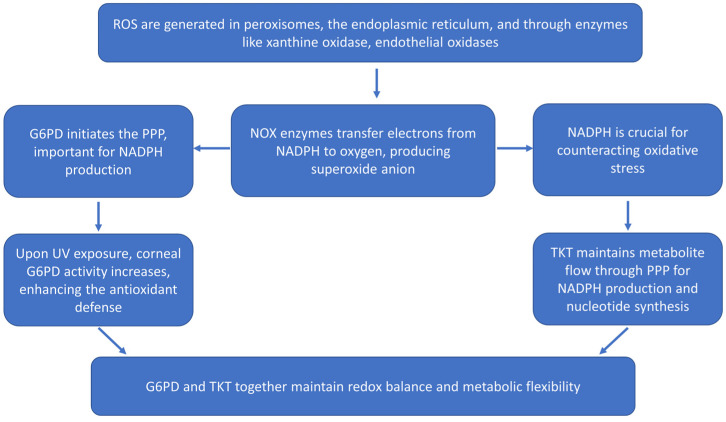
Sequence of events for reactive oxygen species (ROS) generation and antioxidant defense. Glucose-6-phosphate dehydrogenase (G6PD), NADPH oxidase (NOX), pentose phosphate pathway (PPP), transketolase (TKT).

In eukaryotic cells, oxidative metabolism primarily occurs within the mitochondria. Mitochondria, which are significant sources of ROS and susceptible to RNS exposure, are essential for maintaining cellular homeostasis [10]. Cellular respiration within mitochondria takes place along the electron transport chain, where oxygen (O_2_) is reduced to produce adenosine triphosphate (ATP) through oxidative phosphorylation, forming high-energy phosphate bonds [4,5,11]. ROS are additionally generated in peroxisomes, the endoplasmic reticulum, and through reactions catalyzed by xanthine oxidase, endothelial oxidases, and NADPH oxidase (NOX) in phagocytic cell membranes [12,13,14]. The NOX family of enzymes functions as transmembrane carriers, facilitating the transfer of an electron from cytosolic NADPH to oxygen, leading to the reduction of oxygen to superoxide anion [14]. Glucose-6-phosphate dehydrogenase (G6PD) initiates the pentose phosphate pathway (PPP), a crucial metabolic route for generating NADPH [15]. NADPH serves as a key source of reductive power, crucial for counteracting oxidative stress [16]. Upon exposure to ultraviolet light (UVA) or low doses of UVC, corneal G6PD activity increases. This boost in activity could be significant for the cornea’s antioxidant defense against oxidative stress caused by ultraviolet light [17]. Transketolase (TKT) maintains the flow of metabolites through the PPP, ensuring the availability of substrates for both NADPH production and nucleotide synthesis [18]. Together, G6PD and TKT play complementary roles in sustaining cellular redox homeostasis and metabolic flexibility, which are critical for cell survival and function under various physiological conditions, as illustrated in Figure 1.

Due to their high reactivity, ROS can interact with proteins, lipids, and nucleic acids at elevated concentrations, causing functional alterations or destructive effects [19]. The ocular surface, including the tear film, cornea, and aqueous humor, forms the eye’s primary physical and biochemical defense against free radicals [20]. However, the entire surface of the eye, including the tear film, cornea, anterior sclera, aqueous humor, lens, and retina (both the neurosensory retina and pigment epithelium), is highly susceptible to oxidative stress. These areas are especially vulnerable to UV radiation, a major source of ROS [11,21]. Positioned at the front of the eye, the cornea is persistently exposed to high levels of oxygen and solar UV radiation, resulting in the chronic accumulation of ROS [9,11]. Continuous UV exposure, along with environmental pollution, viral infections, chemical injuries, drug intake, and smoking, generates ROS that react with proteins and lipids, leading to lipid peroxidation and significant damage to cell membranes [9,22].

The resilience of healthy corneal epithelial tissue is due to its abundant antioxidant enzymes, which play a crucial role in neutralizing free radicals. The corneal antioxidant defense system encompasses both enzymatic and nonenzymatic antioxidants, such as retinol (vitamin A), ascorbic acid (vitamin C), α-tocopherol (the most active form of vitamin E), GSH, ferritin, CAT, SOD, GPX, Prx, GR, and G6PD [11,21,22]. Additionally, certain members of the aldehyde dehydrogenase (ALDH) superfamily, which are abundantly expressed in the cornea, are recognized for their antioxidant properties [21]. The corneal ALDH plays a vital role in detoxifying cytotoxic aldehydes resulting from membrane lipid peroxidation [3]. Enzymes such as CAT, GPX, Prx, and ALDH are the primary components of the enzymatic antioxidant defense system of the anterior sclera [22]. The tear film and aqueous humor play crucial roles in the ocular surface’s defense mechanisms. SOD is the only reported antioxidant enzyme in the tear film [21]. The antioxidant profile of the aqueous humor closely resembles that of the tear film. In human aqueous humor, the predominant nonenzymatic antioxidants are ascorbic acid, L-tyrosine, uric acid, L-cysteine, and GSH [21]. The absence of blood vessels in the lens heightens oxidative stress [22]. The primary nonenzymatic antioxidant in the lens is reduced GSH [11]. Other significant antioxidant components include ascorbic acid, GPX, SOD, ALDH, and all Prx isoforms, forming a robust endogenous defense mechanism against various oxidative damages [21,22]. CAT mRNA expression is present throughout the lens, with some studies showing higher concentrations at the lens epithelium’s periphery, where cells may require a stronger defense system [22]. Additionally, metabolic enzymes involved in NADPH regeneration, such as GAPD and TKT, are crucial components of the lens antioxidant defense system [22].

**Figure 2:**
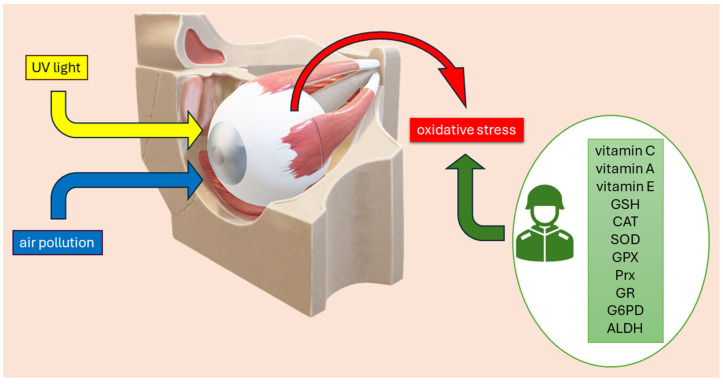
The impact of environmental factors on oxidative stress in the eye and the role of antioxidants. Ultraviolet (UV) light and air pollution contribute to oxidative stress, leading to structural and functional damage in ocular tissues. The eye’s defense mechanisms include both enzymatic and nonenzymatic antioxidants such as Vitamin C, Vitamin A, Vitamin E, glutathione (GSH), catalase (CAT), superoxide dismutase (SOD), glutathione peroxidase (GPX), peroxiredoxins (Prx), glutathione reductase (GR), glucose-6-phosphate dehydrogenase (G6PD), and aldehyde dehydrogenase (ALDH), which help neutralize reactive oxygen species (ROS) and maintain cellular redox balance.

The posterior ocular segment, which includes the optic nerve and retina, has higher metabolic rates and oxygen consumption compared to other body tissues. As a result, more ROS are generated in the mitochondria of these neuronal structures. This damage ultimately leads to the death of retinal ganglion cells and the optic nerve [1]. Due to low levels of antioxidant enzymes and a high content of oxidizable structures, these areas are particularly susceptible to oxidative damage [11]. Primary enzymes such as SOD, GPX, CAT, and Prx are the main components of the enzymatic antioxidant defense system in the retina and retinal pigment epithelium (RPE). Additional antioxidants such as GAPD and certain selenoproteins support the functions of SOD, GPX, Prx, and CAT enzymes [22]. One of the most significant consequences of oxidative stress is the enhancement of lipid peroxidation, leading to the oxidation of polyunsaturated fatty acids in cellular membranes [23]. The products of this process include conjugated dienes and lipid peroxides, as well as secondary products of lipid peroxidation such as malondialdehyde (MDA), 4-hydroxy-2-nonenal [24], and isoprostanes [25]. The retina, rich in PUFA-containing membranes, is highly susceptible to lipid peroxidation triggered by ROS, leading to significant retinal dysfunction and cell death. MDA has been detected in ocular tissues and fluids in experimental models of retinal diseases and serves as a biomarker for oxidative stress in human ocular media [2]. The significant role of environmental factors in contributing to oxidative stress and the protective functions of various antioxidants in the eye are illustrated in Figure 2.

## 3. Impact of Trace Elements on Oxidative Stress

Iron is indispensable for ocular health, playing roles in oxygen transport, enzymatic activity, cellular energy production, neuronal metabolism, oxidative phosphorylation, myelin production, and the synthesis of neurotransmitters [26]. In biological systems, ferrous ions (Fe^2^⁺) and hydrogen peroxide (H_2_O_2_) engage in the Fenton reaction, producing highly reactive hydroxyl radicals (^•^OH) and ferric ions (Fe^3^⁺). This reaction makes free iron a source of ROS, contrasting with protein-bound iron, which exhibits antioxidant properties. The hydroxyl radical is very reactive and can cause oxidative damage to lipids, DNA, and proteins [27]. Given the retina’s lipid-rich environment and high metabolic activity, it is particularly vulnerable to oxidative damage induced by ROS generated through processes such as the Fenton reaction. Retinal exposure to oxidative stress is exacerbated by its high oxygen consumption, making it prone to damage from ^•^OH radicals and other ROS [11,28]. Lipid peroxidation, a consequence of ROS activity, compromises membrane integrity and disrupts cellular signaling pathways that are critical for retinal health [29]. Furthermore, oxidative damage to proteins can impair enzyme activities and receptor functions, further contributing to cellular dysfunction. The accumulation of ROS-induced DNA damage can activate cell death pathways, including apoptosis, which is associated with age-related macular degeneration (AMD) [30]. Ferroptosis, an iron-dependent form of cell death characterized by lipid peroxidation, has been increasingly recognized in retinal diseases linked to iron overload, contributing to photoreceptor degeneration and damage to RPE cells [31]. Addressing these damaging effects involves several strategies. One approach is to use iron chelators, such as deferiprone and deferoxamine, to reduce the availability of free iron, thereby limiting the Fenton reaction and lowering ROS production. These chelators not only decrease oxidative stress but also exhibit anti-inflammatory effects by preventing iron-catalyzed pro-inflammatory signals, providing protective benefits to retinal cells [32]. In addition, the regulation of iron homeostasis proteins such as hepcidin and ferritin helps maintain normal iron levels in the retina by reducing free iron availability and preventing iron-induced oxidative damage [33]. Additionally, the regulation of iron homeostasis proteins, such as hepcidin and ferritin, plays a crucial role in maintaining iron levels in the retina. Hepcidin regulates iron export by binding to ferroportin and triggering its internalization and degradation, thus reducing iron availability and limiting oxidative stress. Ferritin, on the other hand, stores excess iron, preventing it from participating in harmful redox reactions and offering protection against iron-induced damage in retinal cells [33]. The heme group, which contains Fe^3^⁺, serves as the catalytic center of all four polypeptide chains in CAT [34]. CAT, an oxidoreductase enzyme, binds iron and catalyzes the conversion of hydrogen peroxide into water [35]. Heme oxygenases, inducible enzymes, act as measurable indicators of oxidative stress. They catalyze the oxidation of cellular heme, resulting in the production of carbon monoxide, biliverdin, and free ferrous iron [36].

Zinc is important in maintaining the health of the retina, given that zinc is an essential constituent of many enzymes and is needed for optimal metabolism in the eye. Zinc ions are present in SOD, which plays an important role in scavenging superoxide radicals [37]. Zinc neutralizes free radicals directly through GSH or indirectly by acting as a cofactor for GPX [38]. It also inhibits NADPH oxidases, which generate singlet oxygen radicals from oxygen using NADPH [38]. It plays a vital role in protein structure and cell membrane integrity, protecting sulfhydryl groups from oxidation and competing with iron and copper ions for binding sites, thereby reducing the generation of ROS [37,39]. Zinc promotes the production of metallothionein, which neutralizes ROS and possesses strong anti-inflammatory and antioxidant properties, including the inhibition of tumor necrosis factor-alpha-induced nuclear factor kappa-light-chain-enhancer of activated B cells [38,40]. Additionally, metallothionein can effectively bind heavy metal ions, including zinc, copper, chromium, cadmium, and mercury [38].

Copper, present in both Cu^+^ and Cu^2+^ forms, is a crucial cofactor for many enzymes involved in electron transfer within metabolic pathways [41]. In cells, copper plays a dual role: it is vital for the function of numerous enzymes, yet an excess amount can cause oxidative stress and lead to cell death. Copper displays both pro-oxidant and antioxidant properties [42,43]. It engages in a Fenton-like redox reaction, resulting in the production of ROS [44]. Copper is a constituent of SOD: Cu/Zn-SOD, which is present in most body cells, including red blood cells, and extracellular (EC)-SOD, a copper-containing enzyme found in high levels in the lungs and low levels in blood plasma [45]. Nearly all of the copper in our bodies is bound to either transport proteins (ceruloplasmin and Cu-albumin), storage proteins (metallothioneins), or copper-containing enzymes [45]. Copper deficiency may reduce selenium-dependent glutathione peroxidase (Se-GPX) activity by decreasing Se-GPX mRNA levels [45].

The homeostasis of iron, zinc, and copper is a tightly interlinked and finely regulated process essential for cellular function and overall health. Iron and copper levels are thought to increase with age, while zinc levels decrease with age. Alterations in biometal homeostasis trigger oxidative stress as biometals constitute essential cofactors of antioxidant enzyme pathways, which might be risk factors for various age-related ocular diseases. Chronic oxidative stress and inflammation feed each other, leading to a vicious cycle of immune system activation and, consequently, increased age-related ocular complications [46].

In its inorganic form, selenium primarily exists as Se^4+^ and Se^6+^ ions [47]. Selenium’s physiological effects are largely attributed to its incorporation into selenoproteins. In humans, there are 25 genes encoding selenoproteins [48]. Among them, selenoprotein O is found in the mitochondria, H and R in the cell nucleus, I and T in the Golgi apparatus, F, I, K, M, N, S, and T in the endoplasmic reticulum, and R, V, and W in the cytoplasm. Only selenoprotein P is present in the plasma [49] and it is produced by the lacrimal gland and secreted in tears [9,50]. The functions and locations of selenoproteins in ocular tissues are summarized in Table 1. Selenium is an essential component of the amino acids selenomethionine (Se-Met) and selenocysteine. Selenocysteine, in particular, is found in the catalytic centers of antioxidant enzymes such as GPX and TrxR, which help protect cells from damage caused by oxidative stress [47,51,52]. GPX protects cells from oxidative damage by reducing peroxides, harmful by-products of cellular metabolism that can damage lipids, proteins, and DNA [53]. GPX1 is the most abundant selenoprotein and a major metabolic form of selenium in the body, protecting against severe oxidative stress [54]. GPX mediates the catalytic reduction of peroxides using GSH, the main cellular antioxidant, as a sacrificial reductant [6]. GSH is the key antioxidant protecting RPE cells from oxidative damage. The synthesis of GSH largely depends on cysteine availability, which is regulated by the cystine-glutamate exchanger (system xc-) [55]. TrxR operates alongside NADPH and Trx within the thioredoxin system, offering antioxidant benefits [56].

## 4. Trace Elements in the Eye

Iron, one of the most abundant metals in the retina and critical for retinal function as an integral component of key retinal enzymes, shows a variable distribution, occurring mainly in the choroid, RPE, and photoreceptor layers [26]. The eye has developed several mechanisms to manage iron and protect against its potential toxicity. Proteins such as ferritin and transferrin sequester free iron, reducing its availability for participation in harmful reactions [26]. Another iron-regulatory protein that plays a crucial role in maintaining retinal iron homeostasis is hepcidin, the main peptide hormone regulating systemic iron [26]. Maintaining mitochondrial iron homeostasis is critical for the normal physiology of the retina. Any imbalance in the redox system within mitochondria has been linked to age-related retinal pathophysiology [26]. During the natural aging process, iron progressively accumulates within the outer retina [5]. One proposed contributor to the destruction of the RPE and the evolution of AMD through oxidative damage is excessive iron in the RPE and photoreceptors [27]. In cases of AMD, elevated levels of ferritin and ferroportin are found in the retina [8]. Increased iron results in enhanced oxidative stress via the generation of free radicals, subsequently leading to free radical-induced cell death [46]. Melanin sequesters iron ions and protects against oxidative damage [27]. Tang et al. [36] employed a combination of in vitro and in vivo methodologies to explore the role of ferroptosis in RPE degeneration associated with AMD. RPE cells were cultured and treated with sodium iodate (NaIO_3_) to induce oxidative stress, and the role of the Nrf2–SLC7A11–HO-1 pathway in mediating ferroptosis was investigated using high-throughput RNA sequencing, biochemical assays for lipid peroxidation and ROS levels, and heme oxygenase 1 (HO-1) inhibition through zinc protoporphyrin IX and gene knockdown techniques. The study’s methods provided a detailed examination of the molecular mechanisms involved, though it acknowledges limitations such as the non-linear relationships between trace element levels and ferroptosis, and the relatively small sample sizes, which suggest the need for further research to fully understand these complex interactions. This study demonstrated that the primary pathological processes during RPE degeneration in a sodium iodate-induced oxidative stress model involve ferroptosis regulated by HO-1. They discovered that knockdown of HO-1 or the use of the HO-1 inhibitor zinc protoporphyrin-9 can inhibit RPE ferroptosis by disrupting the vicious cycle between ferrous ion accumulation and HO-1 upregulation. This ultimately prevents photoreceptor degeneration and protects visual function.

Iron is necessary for many functions within a healthy cornea, and disruptions of iron homeostasis may contribute to the etiology of corneal diseases. A number of eye diseases affect the cornea, and some involve perturbations of the epithelium. Evidence for iron’s potential participation in corneal disease is demonstrated by the development of iron lines in the cornea. Iron lines develop in the normal aging cornea as well as in the keratoconus and pterygium. They can also be seen near filtering blebs following surgery for glaucoma [27]. Iron is implicated in the pathogenesis of cataracts because of its participation in the formation of oxygen free radicals and the fact that iron foreign bodies in the eye cause cataracts [27]. Iron intake recommendations vary by country, often reflecting local dietary habits, nutritional priorities, and public health guidelines. For instance, adult women in the UK require 14.8 mg of iron per day, while the Recommended Dietary Allowance (RDA) in the USA is 18 mg per day [66]. Recommendations for iron intake also vary significantly between European countries, often ranging from 9 to 15 mg/day, depending on the country and demographic group [67]. The Estimated Average Requirement (EAR) or Average Requirement (AR) for iron is typically around 6 mg/day. Recommended serum iron levels for healthy adults range from 60 to 170 μg/dL, with variations based on age, gender, and dietary factors [68,69]. To prevent the risk of iron overload, which may lead to conditions such as hemochromatosis and ultimately cause tissue damage in the liver, heart, and pancreas, the upper tolerable intake (UL) of iron has been set at 45 mg/day [68]. High iron levels have been associated with an increased risk of cardiovascular diseases, type 2 diabetes, and some cancers due to oxidative stress and inflammation [68,70]. Excess iron in the eye may cause photoreceptor dysfunction and affect the inner retina [26]. Excess iron contributes to retinal cellular damage, leading to retinal cell degeneration and potentially worsening conditions such as AMD, diabetic retinopathy, and hereditary retinal disorders [27,71]. On the other hand, iron deficiency can lead to reduced oxygen transport and metabolic activity in the retina, potentially impairing visual function [26]. However, the direct effects of iron deficiency on eye health are less documented compared to iron overload. Additionally, low iron levels can weaken the immune system, making the body more susceptible to infections. During pregnancy, iron deficiency can lead to premature birth, low birth weight, and developmental delays in infants [72].

Zinc is highly concentrated in the choriocapillaris, RPE, and the retina itself, where levels are significantly higher compared to other body tissues and support numerous zinc-binding proteins involved in visual functions and macular health [7,73]. Zinc also interacts with melanin in pigmented tissues and is abundant in nervous tissues, including the neural retina and the associated RPE [74,75]. It influences cell signaling and nerve impulse transmission [37]. Miceli et al. [76], explore the effects of moderate zinc deficiency on antioxidant defenses and oxidative stress in the retina and RPE of Brown Norway rats. Over a six-week period, rats were fed diets with varying zinc levels, revealing that reduced zinc intake significantly decreased metallothionein levels in the retina and RPE, leading to a marked increase in oxidative stress, as evidenced by elevated thiobarbituric acid reactive substances (TBARS). While retinal zinc content dropped notably in the zinc-deficient group, no significant changes were observed in the RPE-choroid zinc levels, suggesting a differential tissue response. The study highlights the critical role of zinc in maintaining retinal health, particularly through metallothionein’s function in protecting against lipid peroxidation, and suggests that even moderate zinc deficiency could exacerbate oxidative damage in the retina, with implications for AMD. However, the study’s methodology has limitations, including the lack of detailed discussion on whether the relationship between zinc levels and oxidative stress was linear, curvilinear, or asymptotic, as well as the potential impact of sample size on the confidence in outcomes.

In healthy adults, serum zinc levels typically range from 60 to 120 µg/dL [68]. Recommended daily zinc intake varies with factors such as age and gender, typically ranging from 8 to 11 mg/day, and may be dependent on the amount of phytate in the diet [68]. Due to dietary habits, vegetarians, who typically absorb less zinc, may need up to twice as much as meat eaters [37]. The EAR or AR of zinc also varies, typically ranging from 6.5 to 12 mg/day. Zinc concentration is crucial [75]. Zinc deficiency not only increases oxidative stress in the retina but also leads to the accumulation of lipopigment in the aging retinal pigment epithelium [37,74]. Symptoms of zinc deficiency can include night blindness and visual impairment, which can be improved with zinc supplementation [8,75]. In addition, zinc has anti-inflammatory properties, and deficiency can lead to chronic inflammation, a risk factor for various chronic diseases [68]. While zinc is essential for immune function, excessive zinc intake can have the opposite effect, potentially suppressing the immune response and increasing susceptibility to infection [68]. To protect against the potential toxicity of excessive zinc intake, the UL is set at 40 mg/day. The upper limit represents the maximum daily intake at which adverse health effects are unlikely to occur in the general population [68]. High levels of zinc can interfere with copper absorption, leading to copper deficiency, which can cause anemia, neurological symptoms, and other health problems [77].

Zinc plays a crucial role in antioxidant defense, potentially offering protection against oxidative stress, which is a known contributor to eye diseases such as cataracts, diabetic retinopathy, and AMD [78]. Ozdemir et al. [79] investigate the protective effects of zinc supplementation against remote ocular injury caused by intestinal ischemia-reperfusion (IR) in rats. The researchers divided 40 male Sprague Dawley rats into four groups: a control group, a zinc-sham group, an IR group, and a Zn-IR group. IR was induced by clamping the superior mesenteric artery for one hour, followed by 90 min of reperfusion. Zinc aspartate (50 mg/kg) was administered intraperitoneally 15 min before reperfusion in the Zn-IR group. The study measured oxidative stress markers, including MDA, nitric oxide (NO), SOD, and CAT in the chorio-retinal tissue. The results showed that IR significantly increased oxidative stress in the eye, as indicated by elevated MDA levels, which were markedly reduced in the Zn-IR group, suggesting that zinc mitigated the oxidative damage. Zinc treatment also lowered NO levels, though it did not significantly affect the antioxidant enzyme activities of SOD and CAT. In environments where NO is present, free radicals can create peroxynitrite (ONOO^−^), which, together with the direct effects of oxygen radicals, amplifies oxidative damage. Tissue damage and elevated nitric oxide levels may correlate with reduced zinc and SOD activity. Furthermore, zinc can influence cytokines to limit nitric oxide production and lessen oxidative stress byproducts. The findings suggest that zinc supplementation may protect against remote ocular injury by reducing oxidative stress, although the precise mechanisms, particularly the role of antioxidant enzymes, require further investigation. The study highlights the potential clinical significance of zinc in protecting the eye from oxidative damage in conditions associated with systemic IR injury.

Zinc deficiencies and decreased antioxidative capacity are linked to retinal diseases such as AMD, which result from a combination of aging, genetic factors, environmental influences, and oxidative stress [7,8,75]. Zinc levels in the retina and RPE decline with age, correlating with AMD progression. Lower zinc levels in the eyes of AMD patients suggest that zinc deficiency might exacerbate oxidative stress and retinal damage [8,80]. Ha et al. [81] show that zinc induces the ARE-Nrf2 pathway in RPE cells, leading to increased antioxidant and detoxification responses. This molecular mechanism is likely responsible for the observed beneficial effects of zinc in treating AMD.

Mano et al. [82] evaluate the effects of 12 weeks of oral zinc acetate dihydrate (50 mg/day) supplementation on markers of oxidative stress in patients with early AMD. Zinc supplementation led to a notable improvement in oxidative stress markers in patients with early AMD. MDA levels showed a significant reduction, and SOD levels significantly increased. The study also observed a reduction in the area of soft drusen, suggesting that zinc treatment may affect the drusen phenotype in early AMD. The researchers concluded that oral zinc acetate dihydrate (50 mg/day) supplementation could be beneficial in managing early AMD by modifying oxidative stress levels and potentially reducing drusen size. Zinc treatment enhances the antioxidant profile of RPE cells and offers significant cytoprotective effects against oxidative stress.

The findings suggest that while zinc supplementation is beneficial, particularly in high-risk populations such as the elderly with low dietary antioxidant intake or those exposed to cadmium, its effectiveness in inducing specific antioxidant responses such as the expression of heme oxygenase-1 (HO-1) is limited compared to manganese. This could guide future therapeutic strategies where a combination of zinc and manganese might be considered to enhance the antioxidant defenses of the retina more effectively, especially in conditions characterized by high oxidative stress such as AMD [78]. Zinc cysteine, in particular, provides superior protection and antioxidant benefits over other zinc salts or cysteine alone [82]. Satyam et al. [83] investigate the efficacy of a combined formulation of grape seed extract and Zincovit tablets in preventing the onset and progression of age-related cataracts induced by sodium selenite in juvenile Wistar rats. The research demonstrated that sodium selenite significantly increased oxidative stress, leading to cataract formation, which was mitigated in a dose-dependent manner by the combined treatment, as evidenced by reduced MDA levels and restored GSH levels in the lens. Biochemical analyses further revealed that levels of oxidative stress markers such as G6PD and ATP were significantly higher in the treated group compared to the control groups, indicating better preservation of lens function and cellular metabolism. While the treatment also enhanced the activities of antioxidant enzymes, contributing to the protective effect, the study’s methodology has limitations. Specifically, it did not fully explore whether the relationship between trace element levels and oxidative stress was linear, curvilinear, or asymptotic, raising questions about the optimal dosage and response variability. Additionally, the confidence in the outcomes is limited by the sample size and specific experimental conditions, indicating a need for further research with larger sample sizes and more detailed dose–response analyses to validate the therapeutic potential of this formulation.

Both severe copper deficiency and excess are linked to elevated production of ROS and cell death resulting from mitochondrial dysfunction. However, even minor, non-toxic fluctuations in cellular copper levels can influence cell growth and differentiation by modifying mitochondrial metabolism. This modulation can shift the balance between glycolysis and oxidative phosphorylation, as well as alter ROS production, leading to an oxidative cellular environment [84,85]. Copper plays a vital role in the functioning of the retina and is essential for antioxidant defense mechanisms. These mechanisms are important for the survival of the retina since this tissue is routinely exposed to high levels of oxidative stress from light and metabolic processes [7]. The RDA for copper ranges from 200 to 900 mg/day, depending on the age of the individual and the specific population group. However, due to limitations in available biomarkers and balance studies, no EAR or AR for copper has been established. To address this gap, the adequate intake for copper has been established at 1.3 to 1.6 mg/day. This value is intended to cover the needs of most individuals in the absence of a more precise EAR. At the upper end of the range, the UL for copper ranges from 1.0 to 10.0 mg/day, which is the maximum daily intake that is unlikely to cause harmful effects for most people [68]. The free serum copper levels range between 10–15 µg/dL [68]. Copper is necessary for the visual cycle and photoreceptor survival [7]. Copper deficiency has been associated with optic neuropathy, highlighting the importance of adequate copper levels for maintaining retinal and optic nerve health [7,8]. Human disorders of copper metabolism, such as Menkes disease and Wilson disease, result in retinal degeneration, possibly from the loss of copper-transporting proteins in the retinal pigment epithelium [7]. Copper is necessary for the action of tyrosinase, which synthesizes melanin, acting as a storage protein for iron and zinc [8,46]. Melanin is a highly heterogeneous and complex polymer typically found in the pigment epithelium of the iris, choroid, and retina [39].

It was found that the level of metallothioneins decreases with age, causing a change in the cellular dynamics of zinc and copper [46]. Copper deficiency results in a deficiency of zinc [46]. Additionally, decreased levels of copper and zinc with age may be due to increased levels of iron, as they compete for the same ligand and displace each other from receptors. This is further supported by earlier studies in the brain and liver, where investigators found that accumulation of iron following an iron overdose causes depletion in zinc and copper levels [46]. High pigmentation, mainly melanin, is believed to play a protective role for the retina by sequestering heavy metals that might otherwise catalyze undesired oxidative reactions or by trapping free radicals produced by photochemical reactions. In contrast, melanin is also known to produce free radicals and oxidize physiological substrates during ultraviolet and visible light exposure [74]. Additionally, copper is essential for iron metabolism, and its deficiency can lead to anemia due to impaired iron transport and utilization. Copper deficiency has a significant impact on neurodevelopment. Infants with Menkes disease often exhibit severe neurological delays, muscle weakness, and poor motor control [86]. Maintaining balanced copper levels is crucial for brain health. Both deficiency and excess can lead to serious neurological and cognitive problems, emphasizing the importance of proper copper homeostasis in the brain [68]. High levels of dietary copper may also have negative effects, although these are mostly systemic rather than directly ocular. Excessive copper intake is associated with elevated liver enzyme activity and changes in lipid profiles, which may indirectly affect eye health through inflammation or other metabolic disturbances [87].

Retinopathy, a complication of diabetes, can lead to significant vision loss. Bardak et al. [88] explore the protective effects of selenium on retinal pigment epithelium (ARPE-19) and retinal microvascular endothelial cells (ACBRI 181) subjected to high glucose (HG)-induced oxidative stress, a condition relevant to diabetic retinopathy. The researchers used varying concentrations of D-glucose to model HG conditions in vitro and assessed the impact of selenium on several key indicators of cellular stress and apoptosis, including intracellular calcium levels, mitochondrial membrane potential, caspase-3 and -9 activation, ROS production, lipid peroxidation, GSH levels, GPX activity, and vascular endothelial growth factor (VEGF) levels. The results demonstrated that selenium effectively counteracted HG-induced oxidative stress, reducing ROS production, stabilizing mitochondrial function, and decreasing apoptosis rates, while also normalizing VEGF levels and enhancing antioxidant defenses. However, the study’s methodology has limitations, including a lack of detailed exploration of whether the relationship between selenium levels and oxidative stress was linear, curvilinear, or asymptotic, which raises questions about the optimal dosing strategy and potential response variability. Additionally, the confidence in the outcomes may be affected by the specific experimental conditions and the relatively small sample sizes, suggesting the need for further research with larger sample sizes and a more detailed analysis of the dose–response relationship to fully validate the therapeutic potential of selenium in managing diabetic retinopathy.

In the study by Ananth et al. [55], the authors examine the role of Se-Met, an organic form of selenium, in enhancing the antioxidant capacity of RPE cells through the induction of the cystine/glutamate exchanger system xc-. The research emphasizes the importance of GSH in protecting RPE cells against oxidative stress, which is a key factor in AMD. Se-Met was found to significantly induce the expression of the xc- transporter subunit SLC7A11 via activation of the Nrf2 pathway, leading to increased GSH synthesis and enhanced cellular antioxidant defenses. This effect was demonstrated in both ARPE-19 cells and primary mouse RPE cells, where Se-Met treatment improved transporter density and substrate affinity, thereby boosting GSH levels and protecting cells from oxidative stress-induced GSH depletion. However, the study’s methodology has limitations, particularly in its lack of detailed exploration of whether the relationship between Se-Met levels and antioxidant responses was linear, curvilinear, or asymptotic. In addition, the certainty of the results may be limited by the sample size and specific experimental conditions. Nevertheless, the studies suggest that Se-Met has potential as an antioxidant therapy for AMD, although further studies are needed to fully understand its therapeutic effects and determine its appropriate clinical use. Administering selenium to a patient can enhance selenoprotein expression, which helps mitigate the impact of ROS by boosting antioxidant enzyme levels, thereby slowing the progression of chronic ocular disorders [9].

Yazici et al. [89] investigate the retinoprotective effects of selenium in a rat model of retinal ischemia-reperfusion injury, comparing the efficacy of pre-treatment and post-treatment administration. Male Wistar albino rats were divided into four groups: selenium pre-treatment, selenium post-treatment, sham, and control. Selenium was administered as sodium selenite (0.5 mg/kg) intraperitoneally before or after IR injury, with outcomes assessed through biochemical markers such as SOD, GSH, total antioxidant status, MDA, and DNA fragmentation, along with histological evaluations of retinal thickness and apoptosis. The results demonstrated that pre-treatment selenium was more effective in reducing oxidative stress markers, preventing retinal apoptosis, particularly in the ganglion cell layer, and preserving retinal structure compared to post-treatment selenium. While the study provides valuable insights into selenium’s protective role, further research is needed to explore the dose–response relationship and to validate these findings in larger, more diverse sample populations.

The RDA for selenium is set at 55 mg/day for most adults. The EAR or AR for selenium is generally around 45 micrograms per day [68]. Low serum selenium levels (below 70 µg/L) have been associated with cataract formation [9]. Uncontrolled intake of selenium-enriched products may lead to poisoning. Thus, selenium is a trace element with a very narrow range between deficiency, optimal physiological levels, and toxicity [54]. Excessive selenium is linked to various human diseases, including type 2 diabetes, high-grade prostate cancer, amyotrophic lateral sclerosis, and Parkinson’s disease. It may also increase the risk of cataracts, glaucoma, ocular hypertension, and damage to the conjunctiva and cornea [90]. It is important to note that selenium doses above 400 µg/day can be harmful. The recommended serum selenium levels in humans range from 78.9 µg/L to 94.7 µg/L, which are optimal for the function of GPX and other selenoproteins [9].

Gao et al. [90] investigate the toxic effects of excessive selenium, specifically Se-Met, on eye development in zebrafish embryos. The researchers treated zebrafish embryos with 0.5 µM Se-Met and observed significant ocular defects, including microphthalmia, disrupted expression of genes involved in retinal neurogenesis, ectopic cell proliferation, altered mitochondrial morphology, and increased rates of apoptosis and ferroptosis. Notably, antioxidants such as GSH and N-acetylcysteine, as well as the ferroptosis inhibitor ferrostatin-1, were unable to mitigate the Se-Met-induced eye defects. However, the apoptosis activator cisplatin was effective in alleviating these abnormalities, suggesting a complex interplay between apoptosis and ferroptosis in Se-Met toxicity. The study also found a significant increase in ROS and oxidative stress markers in Se-Met-treated embryos, contributing to the observed cellular damage.

DED is a prevalent condition in eye clinics and is closely linked to inflammation and oxidative stress. An innovative formulation of AF127 hydrogel eye drops incorporating copper and selenium nanoparticles (Cu2-xSe NP) has been developed. These nanoparticles act as SOD and GPX, effectively removing ROS and reducing oxidative damage. In a mouse model, the Cu2-xSe NP demonstrated significant therapeutic effects, including antioxidant, anti-apoptotic, and anti-inflammatory properties by modulating NRF2 and p38 MAPK signaling pathways [91].

**Figure 3:**
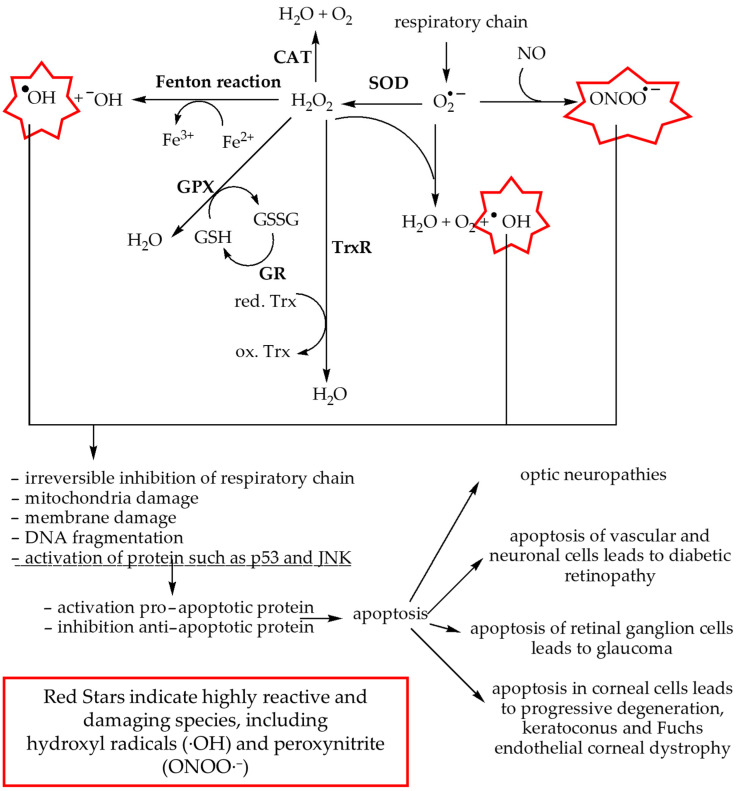
Mechanisms of ROS formation in the context of factors leading to eye diseases. In the respiratory chain, the superoxide anion (O_2_^−^) is generated, which can be converted into hydrogen peroxide (H_2_O_2_) by superoxide dismutase (SOD) or into water (H_2_O) and oxygen (O_2_), potentially leading to the formation of hydroxyl radicals (∙OH). Hydrogen peroxide can be eliminated through several pathways: it can produce hydroxyl anions and OH via the Fenton reaction; it can be converted into water and oxygen in a reaction catalyzed by catalase (CAT); or it can be reduced to water by glutathione peroxidase (GPX) using glutathione (GSH) as a substrate. The oxidized form of glutathione (GSSG) is then reduced back to GSH by glutathione reductase (GR). Another detoxification pathway involves the thioredoxin reductase (TrxR) system, which also leads to the production of water. This reaction involves substrates such as NADPH and thioredoxin (Trx), where oxidized thioredoxin (ox.Trx) is reduced to its active form (red.Trx). Additionally, the superoxide anion can react with nitric oxide (NO) to form peroxynitrite (ONOO^−^). The ROS generated through these reactions (∙OH, O_2_^−^, and ONOO^−^) cause extensive cellular damage, including mitochondrial dysfunction, membrane damage, DNA fragmentation, inhibition of respiratory chain proteins, and the activation of signaling pathways that lead to apoptosis. These processes contribute to a range of diseases, including various eye disorders.

Figure 3 illustrates how ROS are formed and contribute to cellular damage, leading to various eye diseases. These processes involve multiple pathways that result in oxidative stress, which damages cellular components such as mitochondria and triggers apoptosis. This oxidative damage plays a key role in the development of several eye disorders [22,45,55,66,72,92].

**Figure 4:**
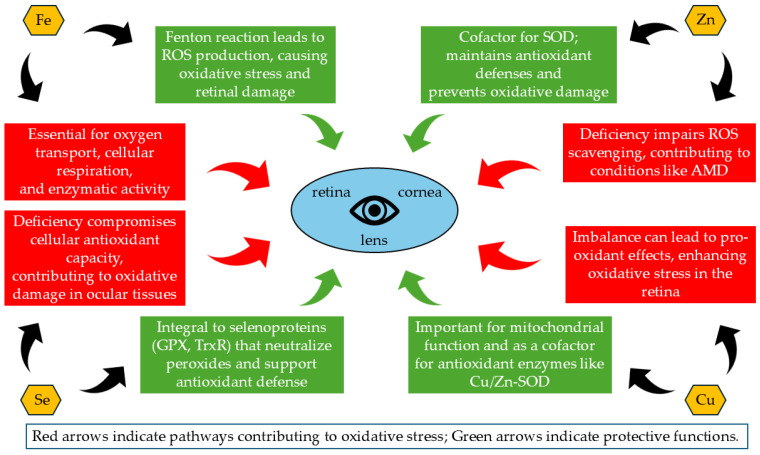
The roles of key trace elements in maintaining ocular health and their contributions to oxidative stress and disease prevention. Iron, Zinc, Copper, and Selenium are critical for enzymatic activity, redox balance, and overall cellular function in the eye. Their dysregulation can lead to various ocular pathologies. (AMD) age-related macular degeneration; (GPX) glutathione peroxidase; (ROS) reactive oxygen species; (SOD) superoxide dismutase; (TrxR) thioredoxin reductase.

The intricate interplay between trace elements and oxidative stress significantly impacts ocular health. Understanding the roles and effects of key trace elements is crucial for developing effective therapeutic strategies to mitigate oxidative damage in eye tissues. Table 2 provides a comprehensive overview of the primary functions, antioxidant mechanisms, and effects of deficiencies and excesses of these essential trace elements, as well as their associations with common ocular diseases. Figure 4 graphically summarizes the roles of key trace elements in maintaining ocular health, highlighting both their protective and detrimental effects on oxidative stress regulation and disease prevention.

## 5. Conclusions

Oxidative stress plays a critical role in the development of various ocular degenerative diseases, leading to both structural and functional alterations in eye tissues. Enzymatic and nonenzymatic antioxidants are essential in maintaining ocular health by neutralizing ROS and restoring cellular redox balance. Key trace elements, including iron, zinc, copper, and selenium, are vital for the proper functioning of these antioxidant systems. The balance of these elements is integral to ocular health, and disturbances can result in significant pathologies such as AMD, cataracts, and glaucoma.

While antioxidant supplementation targeting these trace elements shows promise in mitigating oxidative damage and improving eye health, there remains a significant gap in understanding the precise mechanisms through which these elements interact with the antioxidant systems in the eye. The relationships between trace element levels and their effects on oxidative stress are not yet fully understood, with some studies indicating curvilinear or asymptotic effects. The optimal levels and combinations of trace elements for therapeutic efficacy remain unclear, and more research is needed to determine how best to restore balance and prevent disease progression.

Current research also has limitations, including small sample sizes, variability in methodologies, and a lack of long-term studies on the effects of trace element supplementation in humans. Additionally, while animal models have provided valuable insights, there is a need for more clinical trials to validate these findings in human populations. The potential for adverse effects with excessive supplementation must also be considered, as certain trace elements, such as iron, can exhibit pro-oxidant activity at higher concentrations.

Future research should focus on elucidating the precise mechanisms by which these trace elements regulate oxidative stress and their interactions with various antioxidant systems. Studies investigating combination therapies that utilize multiple trace elements or antioxidants may provide new avenues for enhancing ocular protection. Additionally, exploring the role of dietary interventions tailored to specific populations and the development of targeted antioxidant therapies could offer significant potential for the prevention and management of oxidative stress-related eye diseases.

A comprehensive understanding of these mechanisms and innovative therapeutic approaches will be essential in advancing clinical outcomes and improving the quality of life for individuals affected by ocular degenerative diseases. Addressing the current knowledge gaps and limitations through well-designed studies will be crucial in developing more effective treatments and preventive strategies.

## Figures and Tables

**Table 1:** Roles of selected selenoproteins in ocular health and their functions in protecting against oxidative stress.

Selenoprotein	Functions in the Eye	References
Selenoprotein P (SELENOP)	Transports selenium to the cornea, protecting it from oxidative stress; elevated levels in tear fluid help maintain ocular surface health, particularly in dry eye syndrome.	[50,57,58]
Selenoprotein M (SELENOM)	Contributes to redox regulation and may play a role in protecting the lens from oxidative damage, although specific evidence for tissue expression is not yet available.	[59]
Selenoprotein F (SELENOF)	Involved in protein folding and antioxidant protection in ocular tissues. Dysregulation is linked to cataract development, potentially due to protein misfolding in the endoplasmic reticulum.	[60,61]
Selenoprotein O (SELENOO)	Functions as a mitochondrial redox regulator, potentially protecting retinal cells from oxidative damage. Its role in the eye is still speculative, with more research needed.	[62]
Glutathione Peroxidases (GPX1 and GPX4)	Critical for reducing oxidative damage in the lens and retina. GPX1 deficiency is associated with cataract formation, while GPX4 helps prevent lipid peroxidation in retinal cells and lens tissues.	[9,63]
Thioredoxin Reductase (TxnRd)	Helps maintain redox balance in tissues, protecting them from oxidative stress and preventing damage caused by reactive oxygen species, although specific evidence for its role in ocular tissues is not provided.	[64]
SelJ	Expressed in the eye lens of certain fish species, suggesting a potential structural role. While not found in humans, it highlights the diversity and plasticity of selenoproteins in the eye across species.	[65]

**Table 2:** Roles and effects of key trace elements on oxidative stress and ocular health.

Trace Element	Primary Functions	Antioxidant Mechanisms	Effects of Deficiency	Effects of Excess	Associated Ocular Diseases	References
Iron (Fe)	Oxygen transport, enzymatic activity, cellular energy production, retinal function	CAT activity, Fenton reaction prevention	Anemia, retinal degeneration, impaired visual function	Oxidative stress, photoreceptor dysfunction, AMD progression	AMD, cataracts, retinal degeneration	[5,8,26,27]
Zinc (Zn)	Enzyme constituent, protein structure, cell membrane integrity	SOD activity, metallothionein production	Night blindness, impaired vision, increased oxidative stress	Toxicity, disrupted copper absorption, potential neurotoxicity	AMD, diabetic retinopathy, cataracts	[7,8,37,74,75,76,78,79,80,81,82]
Copper (Cu)	Enzyme cofactor, electron transfer, photoreceptor survival	SOD activity, ceruloplasmin function	Optic neuropathy, impaired photoreceptor function	Oxidative stress, mitochondrial dysfunction, cell death	Wilson’s disease, Menkes disease, retinal degeneration	[7,8,41,42,43,44,45]
Selenium (Se)	Selenoprotein synthesis, antioxidant enzyme function	GPX activity, TrxR function	Increased oxidative stress, susceptibility to infections	Selenosis, increased risk of diabetes, cardiovascular diseases	Cataracts, glaucoma, AMD, ocular surface damage	[9,47,50,51,52,53,54,55,56,89,90,91]

Abbreviations used: AMD: Age-related macular degeneration; CAT: Catalase; GPX: Glutathione peroxidase; SOD: Superoxide dismutase; TrxR: Thioredoxin reductase.
